# Multi-locus sequence typing of *Salmonella enterica *subsp. *enterica *serovar Enteritidis strains in Japan between 1973 and 2004

**DOI:** 10.1186/1751-0147-53-38

**Published:** 2011-06-15

**Authors:** Tamie Noda, Koichi Murakami, Tetsuo Asai, Yoshiki Etoh, Tomoe Ishihara, Toshiro Kuroki, Kazumi Horikawa, Shuji Fujimoto

**Affiliations:** 1 Division of Pathology and Bacteriology, Fukuoka Institute of Health and Environmental Sciences, 39 Mukaizano, Dazaifu, Fukuoka 818-0135, Japan; 2 National Veterinary Assay Laboratory, Ministry of Agriculture, Forestry and Fisheries, 1-15-1 Tokura, Kokubunji, Tokyo 185-8511, Japan; 3 Department of Microbiology, Kanagawa Prefectural Institute of Public Health, 1-3-1 Shimomachiya, Chigasaki, Kanagawa 253-0087, Japan; 4 Department of Health Sciences, Faculty of Medical Sciences, Kyushu University, 3-1-1 Maidashi, Higashi-Ku, Fukuoka 812-8582, Japan

## Abstract

*Salmonella enterica *subsp. *enterica *serovar Enteritidis (*S*. Enteritidis) was responsible for a worldwide pandemic during the 1980s and 1990s; however, changes in the dominant lineage before and after this event remain unknown. This study determined *S*. Enteritidis lineages before and after this pandemic event in Japan using multilocus sequence typing (MLST). Thirty *S*. Enteritidis strains were collected in Japan between 1973 and 2004, consisting of 27 human strains from individual episodes, a bovine strain, a liquid egg strain and an eggshell strain. Strains showed nine phage types and 17 pulsed-field profiles with pulsed-field gel electrophoresis. All strains had homologous type 11 sequences without any nucleotide differences in seven housekeeping genes. These MLST results suggest that *S*. Enteritidis with the diversities revealed by phage typing and pulsed-field profiling has a highly clonal population. Although type 11 *S*. Enteritidis may exhibit both pleiotropic surface structure and pulsed-field type variation, it is likely to be a stable lineage derived from an ancestor before the 1980s and/or 1990s pandemic in Japan.

## Findings

*Salmonella enterica *subsp. *enterica *serovar Enteritidis (*S*. Enteritidis) was responsible for a worldwide pandemic during the 1980s and 1990s; however, changes in the dominant lineage before and after this event remain unknown. It is also difficult to explain why multiple clones of *S*. Enteritidis (defined by phage types, (PTs)) infecting chicken reproductive tissues emerged simultaneously in geographically separate countries during this pandemic. For example, PT 4 emerged in some European countries during the late 1980s, including England and Wales [1], France [2] and Germany [3], while PT 6 dominated Denmark [4]. PT 8, PT 13a and PT 13 were most common in the USA [5] and in Japan, PT 1 and PT 4 were dominant [6]. To understand the simultaneous emergence of this pathogen, it is necessary to deduce whether these multiple lineages co-instantaneously acquired the ability to colonize chicken reproductive tissues or a single clone that possessed this ability evolved into multiple lineages before the pandemic.

Multi-locus sequence typing (MLST) can be used to accurately identify bacterial lineages [7,8]. The genetic distance between two strains can be quantitatively estimated as allelic differences in the nucleotide sequences of housekeeping or virulent genes among bacterial strains [8]. MLST data have been used in evolutionary and population analyses that estimate recombination and mutation rates and also in the investigation of evolutionary relationships among bacteria classified within the same genus [8]. Using MLST, our study aimed to determine the lineages of *S*. Enteritidis before and after the pandemic during the 1980s and/or 1990s in Fukuoka Prefecture, Japan.

*Salmonella *strains used in this study are listed in Table 1. Human strains from individual food-borne disease outbreaks in 1973-1981 were isolated in the Kanagawa Prefectural Institute of Public Health. Other human strains (isolated from individual outbreaks), the liquid egg strain and the eggshell strain were isolated from different samples at the Fukuoka Institute of Health and Environmental Sciences. The cattle strain was provided by the Livestock Hygiene Service Center. All strains were isolated in Japan between 1973 and 2004 (Table 1). We selected these strains to assess long-term alternation or rotation of *S*. Enteritidis genotypes. The study has been approved by the Fukuoka Institute of Health and Environmental Silences Ethics Committee.

**Table 1:** Phage type, pulsed-field gel electrophoresis profile and sequence type of *Salmonella enterica *subsp

**Strain No**.	Isolation Date	Origin	Foods that caused disease	Phage Type	PFGE Profile	ST^a^
1	1973/5/Uncertain	Human	Unidentified	NT^b^	53	11
2	1980/4/Uncertain	Human	Unidentified	NT	52	11
3	1981/2/Uncertain	Human	Unidentified	NT	55	11
4	1992/9/26	A case of an outbreak	Unidentified	3	21	11
5	1993/6/24	A case of an outbreak	Egg-related	1	22	11
6	1993/8/6	A case of an outbreak	Unidentified	1	23	11
7	1993/9/27	A case of an outbreak	Egg-related	1	24	11
8	1994/6/11	A case of an outbreak	Unidentified	1	25	11
9	1994/6/25	A case of an outbreak	Egg-related	9	26	11
10	1994/10/20	A case of an outbreak	Unidentified	1	27	11
11	1994/10/26	A case of an outbreak	Egg-related	9	28	11
12	1995/6/19	Liquid Egg	-	1	9	11
13	1996/1/Uncertain	Egg-shell sample	-	4	1	11
14	1996/7/19	A case of an outbreak	Egg-related	7	10	11
15	1996/11/1	A case of an outbreak	Not Egg-related	1	18	11
16	1997/6/Uncertain	A case of an outbreak	Egg-related	4	1	11
17	1997/6/10	A case of an outbreak	Unidentified	1	1	11
18	1998/6/24	A case of an outbreak	Egg-related	1	1	11
19	1998/8/21	A case of an outbreak	Unidentified	4	1	11
20	1998/10/7	A case of an outbreak	Egg-related	1	1	11
21	1999/7/29	A case of an outbreak	Unidentified	14b	47	11
22	2000/1/7	A case of an outbreak	Egg-related	6a	10	11
23	2000/7/4	A case of an outbreak	Unidentified	4	1	11
24	2000/7/17	A case of an outbreak	Unidentified	1	1	11
25	2001/6/4	A case of an outbreak	Unidentified	36	1	11
26	2001/9/11	A case of an outbreak	Unidentified	1	51	11
27	2001/12/16	A case of an outbreak	Egg-related	6a	10	11
28	2002/Uncertain	Bovine	-	NT	52	11
29	2004/7/16	A case of an outbreak	Egg-related	1	1	11
30	2004/10/22	A case of an outbreak	Unidentified	14b	1	11

*enterica *serovar Enteritidis strains in Japan from 1973 to 2004.

^a^ST, strain type

^b^NT, not tested

Housekeeping genes, *thrA *(aspartokinase+homoserine dehydrogenase), *purE *(phosphoribosylaminoimidazole carboxylase), *sucA *(alpha ketoglutarate dehydrogenase), *hisD *(histidinol dehydrogenase), *aroC *(chorismate synthase), *hemD *(uroporphyrinogen III cosynthase) and *dnaN *(DNA polymerase III beta subunit) were used for MLST characterization. Each overnight-culture of the strains grown in LB broth (Difco Laboratories, Sparks, MD., USA) was centrifuged at 12,000 rpm for 5 min before the supernatant was discarded. The pellet was resuspended in normal saline (1 ml) and centrifuged at 12,000 rpm for 5 min. The supernatant was discarded and the pellet was resuspended in 200 μl of 0.1 × TE buffer before being boiled for 10 min and centrifuged at 12,000 rpm for 10 min. The supernatant was used as a template for the primary PCR. Primers for the primary PCR were as follows: *thrA *F 5'-GTC ACG GTG ATC GAT CCG GT-3'; *thrA *R 5'-CAC GAT ATT GAT ATT AGC CCG-3'; *purE *F 5'-ATG TCT TCC CGC AAT AAT CC-3'; *purE *R 5'-TCA TAG CGT CCC CCG CGG ATC-3'; *sucA *F 5'-AGC ACC GAA GAG AAA CGC TG-3'; *sucA *R 5'-GGT TGT TGA TAA CGA TAC GTA C-3'; *hisD *F 5'-GAA ACG TTC CAT TCC GCG CAG AC-3'; *hisD *R 5'-CTG AAC GGT CAT CCG TTT CTG-3'; *aroC *F 5'-CCT GGC ACC TCG CGC TAT AC-3'; *aroC *R 5'-CCA CAC ACG GAT CGT GGC G-3'; *hemD *F 5'-ATG AGT ATT CTG ATC ACC CG-3'; *hemD *F1 5'-GAA GCG TTA GTG AGC CGT CTG CG-3'; *dnaN *F 5'-ATG AAA TTT ACC GTT GAA CGT GA-3'; *dnaN *R 5'-AAT TTC TCA TTC GAG AGG ATT GC-3'. Amplifications for all genes were carried out with 2 μl DNA template, 2 μl of 2.5 mM (each) deoxynucleoside triphosphates, 5 μl of 5 × PrimeSTAR Buffer (5 mM Mg^2+^) (Takara Bio Inc., Otsu, Japan), 6.25 pmol of primer, and 0.625 U of PrimeSTAR HS DNA Polymerase (Takara Bio Inc.) in 25 μl reaction mixtures. Reaction conditions involved 30 cycles of: (A) denaturation at 98°C for 10 s, (B) primer annealing at 55°C for 10 s, and (C) extension at 72°C for 1 min. Reactions were performed using a PCR Thermal Cycler SP TP 400 (Takara Shuzo, Co., Ltd., Kyoto, Japan). PCR products were purified using Microcon (Millipore Corporation, Bedford, Mass., USA) and sequenced using an ABI Prism BigDye Terminator v3.1 Cycle Sequencing Kit and an ABI 3730 Genetic Analyzer (Applied Biosystems Japan Ltd., Tokyo, Japan). Primers for sequence reactions were as follows: *thrA*-F 5'-ATC CCG GCC GAT CAC ATG AT-3; *thrA*-R 5'-CTC CAG CAG CCC CTC TTT CAG-3'; *purE*-F 5'-CGC ATT ATT CCG GCG CGT GT-3'; *purE*-R 5'-CGC GGA TCG GGA TTT TCC AG-3'; *sucA*-F 5'-AGC ACC GAA GAG AAA CGC TG-3'; *sucA*-R 5'-GGT TGT TGA TAA CGA TAC GTA C-3'; *hidD *-F 5'-GTC GGT CTG TAT ATT CCC GG-3'; *hisD*-R 5'-GGT AAT CGC ATC CAC CAA ATC-3'; *aroC*-F 5'-GGC ACC AGT ATT GGC CTG CT-3'; *aroC*-R 5'-CAT ATG CGC CAC AAT GTG TTG-3'; hemD-F5'-GTG GCC TGG AGT TTT CCA CT-3'; hemD-R5'-GAC CAA TAG CCG ACA GCG TAG-3'; *dnaN*-F 5'-CCG ATT CTC GGT AAC CTG CT-3'; *dnaD*-R 5'-CCA TCC ACC AGC TTC GAG GT-3'. Sequences were edited and the complementary sense and antisense fragments were aligned using the SeqManII program in the Lasergene software package (DNASTAR, Madison, WI, USA).

Sequences were submitted to the MLST database website (http://mlst.ucc.ie/) and assigned existing or novel allele type numbers and sequence type numbers defined by the database. Composite sequence types (STs) were assigned based on the set of allele types derived from each of the seven loci. Allele sequences for each strain were then concatenated in the order *aro*C - *dna*N - *hem*D - *his*D - *pur*E - *suc*A - *thr*A for a final composite length of 3,336 bp. Concatenated sequences were aligned using ClustalW software [9] and a phylogenetic tree was constructed using the neighbor-joining method (Center for Information Biology and DNA Data Bank of Japan) to compare to other published STs. Phylogenetic analyses were performed using NJplot (http://pbil.univ-lyon1.fr/software/njplot.html).

Phage typing was conducted on *S*. Enteritidis strains recovered from samples in this study according to the method of Ward *et al*. [10] at the National Institute of Infectious Disease.

Pulsed-field gel electrophoresis (PFGE) was conducted as described in our previous report [11]. Strains were considered different if variation in one or more DNA bands was observed. Using FPQuest Software (Bio-Rad Laboratories Inc. Hercules, CA, USA), similarity and cluster analyses were performed with Dice coefficients of similarity and an unweighted pair group method using average linkage, respectively.

**Figure 1:**
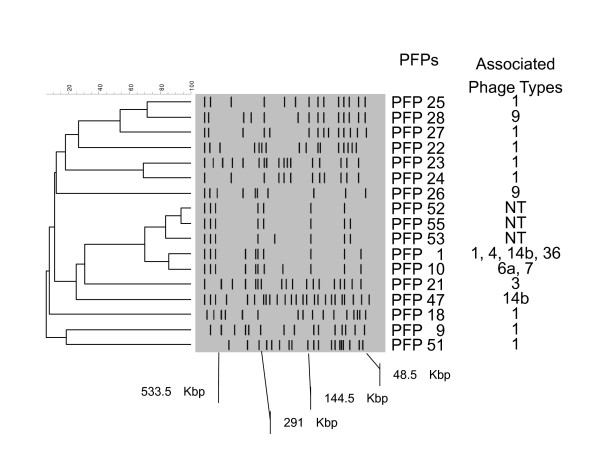
**Pulsed-field gel electrophoresis analysis of fingerprints from genetically defined *Salmonella enterica *subsp**. *enterica *serovar Enteritidis strains. Numbers on the right of the dendrogram indicate pulsed-field profiles (PFPs) and the phage types (PTs) associated with each PFP. The scale indicates the percentage similarity, as determined using Dice coefficients. PFPs 1 and 10 include some strains. The center diagrams are the PFPs of *Salmonella enterica *subsp. *enterica *serovar Enteritidis strains with BlnI digestion.

Phage typing identified nine PTs (Table 1). PT 1 was most dominant, while PT 3, PT 7 and PT 36 were individually detected in only one strain for each. For PFGE analysis, 30 strains generated 17 pulsed-field profiles (PFPs) after restriction with BlnI (Table 1 Figure 1). PFP 1 was most dominant (11 strains), followed by PFP 10 (three strains). All other PFPs were only found in a single strain. *S*. Enteritidis with PFP 1 consisted of PT 1, PT 4, PT 14b and PT 36 strains.

**Figure 2:**
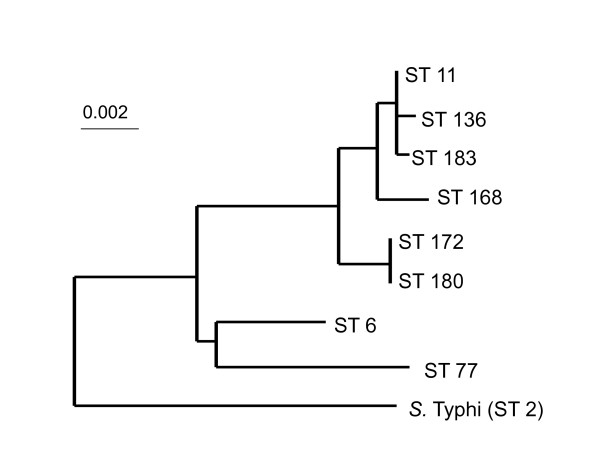
**Phylogenetic tree of *Salmonella enterica *subsp**. *enterica *serovar Enteritidis and *Salmonella enterica *subsp. *enterica *serovar Typhi according to STs from the MLST database (http://mlst.ucc.ie/mlst/dbs/Senterica), showing the difference between ST 11 and others. All strains from our study were ST 11. Allele sequences for each strain were concatenated in the order *aro*C - *dna*N - *hem*D - *his*D - *pur*E - *suc*A - *thr*A for a final composite length of 3,336 bp. Sequences were aligned using the ClustalW method [9] and phylogenetic analyses were performed using NJplot through the Web Search and Analysis Service System provided by DNA Data Bank of Japan (Center for Information Biology and DNA Data Bank of Japan). All strains were designated in ST11. The scale represents nucleotide substitutions per site.

MLST analysis did not detect any nucleotide differences among the 30 *S*. Enteritidis strains for seven genes (*thrA*: 501 bp, *pure*: 399 bp, *sucA*: 501 bp, *hisD*: 501 bp, *aroC*: 501 bp, *hemD*: 432 bp, and *dnaN*: 501 bp). All strains were characterized as ST 11 according to the MLST database (Table 1). The sequences of ST 11 are available in the MLST database http://mlst.ucc.ie/. ST 11 consisted of strains with variable PTs and PFPs. According to concatenated sequences analysis, ST 11 belonged to a clade that consisted of ST 11, ST 136, and ST 183 (Figure 2).

For the first reported time, our study characterized all tested *S*. Enteritidis strains between 1973 and 2004 as a single ST (ST 11) and several PTs and PFPs. Two main interpretations of these results include: (A) Rabsch and colleagues speculated that *S*. Enteritidis acquired a niche in chicken reproductive tissues before the 1980s [12]; (B) disease-associated *S*. Enteritidis had a higher clonal population in Fukuoka Prefecture, Japan. These hypotheses are further discussed below.

Persistence of *S*. Enteritidis clonality in Japan supports the idea that the ST 11 lineage had possibly acquired a niche within chicken reproductive organs before the pandemics (1980s and 1990s). It is reported that the process of chicken oviduct colonization of *S*. Enteritidis is complex and depends on many factors including fimbriae, flagellae, lipopolysaccharide, cell wall structure and stress tolerance [13]. Until now, it has not been clear as to why *S*. Enteritidis is specifically implicated in egg contamination [14]. Housekeeping genes potentially play a less important roll in chicken oviduct colonization than genes of the above-mentioned factors. However, the absence of changes in the housekeeping genes suggests that the ST11 lineage survived without any substantial genetic changes being produced by the fundamental processes of mutation, recombination, selection, drift, and migration [15]. Insufficient information was available for strains isolated before the pandemic because only three isolates were tested. Although ST 11 *S*. Enteritidis may produce some strain variation in pleiotropic surface structures [16] defined by PTs (such as PT 1, 3, 4, 6a, 7, 9, 14b, and 36) and genotypes defined by PFPs, it is likely to be a stable lineage that was derived from an ancestor before the pandemic in Japan. Our study therefore potentially supports the speculative assumptions made by Rabsch *et al*. [12].

Our study also suggested that food-borne disease-associated *S*. Enteritidis has a relatively higher clonal population than other *Salmonella*. In another study, 81 isolates of *S*. Newport from humans, food animals, and retail foods showed 12 STs [17], while in our present study, *S*. Enteritidis populations showed a single ST lineage. As all typing systems are influenced by the tested population [18], we can only discuss human and food related strains in Fukuoka Prefecture, Japan, rather than worldwide *S*. Enteritidis population genetics. However, according to the international MLST database, among the several distinct STs of *S*. Enteritidis in the world, only ST 11 consists of human strains. These results imply that human and food related-S. Enteritidis may be a higher clonal population than *S*. Newport.

MLST is a useful method for analyzing the core genes of pathogens that are of public health importance. MLST can be used to analyze conserved core genes, like housekeeping genes, which are universally conserved and provide fundamental genetic information [19]. Our study has revealed that MLST is suitable for evolutionary, basic relatedness or population studies, however, in contrast with PFGE, this method is not effective for epidemiological purposes. This is because MLST cannot analyze a diverse range of *Salmonella *strain specific genetic information that may be associated with niche adaptation. However, genetic variation in pathogen populations is a major barrier to disease control [20], and therefore, it is pertinent that we continue to survey the *S*. Enteritidis lineage in Japan using MLST.

## Competing interests

The authors declare that they have no competing interests.

## Authors' contributions

TN and KM provided data, discussed the results gained, and drafted the manuscript. TA, YE, TI, TK, KH and SF provided data, discussed the results gained, and participated in revising the manuscript. All authors read and approved the final manuscript.
